# Predictors of Problematic Social Media Use in a Nationally Representative Sample of Adolescents in Luxembourg

**DOI:** 10.3390/ijerph182211878

**Published:** 2021-11-12

**Authors:** Claire van Duin, Andreas Heinz, Helmut Willems

**Affiliations:** 1 Department of Social Sciences, Maison des Sciences Humaines, University of Luxembourg, 11 Porte des Sciences, L-4366 Esch-sur-Alzette, Luxembourg; andreas.heinz@uni.lu; 2 Department of Behavioural and Cognitive Sciences, Maison des Sciences Humaines, University of Luxembourg, 11 Porte des Sciences, L-4366 Esch-sur-Alzette, Luxembourg; helmut.willems@uni.lu

**Keywords:** problematic social media use (PSMU), adolescents, social media, social support, media effects, health behavior in School-aged Children (HBSC), well-being, differential susceptibility to media effects model, preference for online social interaction

## Abstract

Social media use has increased substantially over the past decades, especially among adolescents. A proportion of adolescents develop a pattern of problematic social media use (PSMU). Predictors of PSMU are insufficiently understood and researched. This study aims to investigate predictors of PSMU in a nationally representative sample of adolescents in Luxembourg. Data from the Health Behavior in School-aged Children (HBSC) study in Luxembourg were used, in which 8687 students aged 11–18 years old participated. The data were analyzed using hierarchical multiple regression. A range of sociodemographic, social support, well-being and media use predictors were added to the model in four blocks. The predictors in the final model explained 22.3% of the variance in PSMU. The block of sociodemographic predictors explained the lowest proportion of variance in PSMU compared with the other blocks. Age negatively predicted PSMU. Of the predictors related to social support, cyberbullying perpetration was the strongest predictor of PSMU. Perceived stress and psychosomatic complaints positively predicted PSMU. The intensity of electronic media communication and preference for online social interaction were stronger predictors of PSMU than the other predictors in the model. The results indicate that prevention efforts need to consider the diverse range of predictors related to PSMU.

## 1. Introduction

The last decades have seen a sharp increase in digital technology use and online communication [1,2]. Young people especially have embraced social media, current data suggesting that, on average in Europe, 54% of children and adolescents aged 9–16 use social media at least once daily [1]. The increasing popularity of social media among adolescents has been met with increasing concern about the potential negative effects of social media on the well-being of young people. On the one hand, these concerns have led to research on the effects of social media on the well-being of adolescents. The current scientific knowledge on the relationship between adolescent media use and well-being is complex, often yielding small effect sizes, conflicting findings and resulting in overall knowledge gaps [3,4,5,6]. Previous research has found positive effects of social media on adolescents, such as an increased level of self-esteem and access to online support networks [7,8], as well as negative effects such as an impaired sleep quality and mental health problems [9,10]. It is important to note that the directionality of these effects is still unclear, in particular for well-being. Previous research has found both negative effects of social media on well-being, and of well-being on social media use, emphasizing the importance of considering bidirectional effects [3]. On the other hand, there is increased interest in factors that make adolescents susceptible to media effects, and a need for research on factors that place adolescents at risk of negative effects of social media use [3,11]. Previous studies have indicated a knowledge gap regarding predictors of problematic social media use (PSMU), which are still insufficiently understood, and state that understanding risk factors for PSMU is essential for the advancement of research on social media behavior [12,13]. This study aims to increase the current understanding of predictors of PSMU among adolescents in Luxembourg(An early draft of this paper was presented at the 12th Excellence in Pediatrics conference [14]). To this end, this study uses the Social Media Disorder Scale to measure PSMU, which is based on shared characteristics of social media platforms and, thus, not limited to one social media platform. Predictors of PSMU were selected based on previous research, as well as propositions determined by the theoretical framework of the Differential Susceptibility to Media Effects model (DSMM) and Ecological Systems theory [5,15]. By using a comprehensive set of predictors in one model, we can consider the relative importance of the predictors of PSMU.

### 1.1. Definition of Problematic Social Media Use

A fraction of the adolescents that use social media seem to develop a problematic and compulsive pattern of use called PSMU, also known as social media addiction or problematic social networking use [16]. Due to the lack of a common definition for PSMU, varying assessment tools and possible cultural differences between samples and countries, the prevalence of problematic social media use differs between studies [17]. Recent large-scale studies indicate that the prevalence of PSMU among European adolescents is around 4.5–9.4% [17,18,19]. Social media addiction, in contrast with internet gaming disorder, is at present not recognized as a disorder in the latest edition of the Diagnostic and Statistical Manual of Mental Disorders (5th ed) [20]. However, researchers have put forth the idea that both disorders fall under the overarching construct of internet addiction and can be assessed using the same diagnostic criteria, such as persistence, tolerance, escape and conflict [21,22].

### 1.2. Theoretical Framework

In this paper, the Differential Susceptibility to Media Effects Model and the Bio-Ecological Model were used to select potential factors which may contribute to the risk of problematic social media use among adolescents. The Differential Susceptibility to Media Effects Model (DSMM) was developed by Valkenburg and Peter (2013) to increase our understanding about media effects [5]. Media effects are short-term and long-term changes in the emotion, behavior, cognition, physiology, attitudes and beliefs within a person as a result of media use [5]. The DSMM is focused on microlevel media effects, meaning effects on the individual media user, and builds on previous theories such as the Uses and Gratifications Theory by Katz, Blumler and Gurevitch, Social Cognitive theory by Bandura and the General Aggression Model by Anderson and Bushman [23,24,25]. Although the model was not developed for children and adolescents specifically, it builds on earlier theories used to understand media effects in these groups.

The DSMM reflects the belief that the effect of media on adolescents is diverse. The proposition that is most relevant for this paper is that media effects are conditional on dispositional, developmental and social susceptibility variables, which influence the individual’s responsiveness to and selection of media. Dispositional susceptibility variables are individual characteristics such as gender and moods. Developmental susceptibility variables account for the role of the emotional, social and cognitive development of an individual. Social susceptibility variables are factors that relate to the social context, such as family, school and social norms [5]. The DSMM calls these variables “differential susceptibility variables”, as the factors may increase the individuals susceptibility for negative and positive media effects [5,11].

Media effects and behavior that adolescents engage in online can also be considered from an ecological perspective. From this perspective, the concern about the social media use of adolescents can be understood, seeing that the daily activities adolescents engage in are considered an important influence on their development [26]. The Bio-Ecological Model, first introduced by Bronfenbrenner in 1977, proposes that an individual is influenced by relationships and interaction with their environment. In this paper, we draw inspiration from the Bio-Ecological Model by considering how various individual (e.g., gender), environmental (e.g., family) and social factors (e.g., affluence) affect PSMU. The model divides the environment into several nested structures: the microsystem, the mesosystem, the exosystem and the chronosystem [15,26]. An important aspect of this model is that adolescents are not seen as passive recipients of influences on their activities or development, but that they have an active role in shaping these experiences [26]. This is in accordance with contemporary media effect theories which acknowledge that while media can influence the individual media user, the individuals themselves are the origin for that process of change [6].

### 1.3. Predictors of Problematic Social Media Use

**Sociodemographic Factors**

Studies exist on the relationship between PSMU and sociodemographic characteristics, but the results are contradictory and, in some cases, not comparable. Schivinski et al. (2020) found that age and gender did not significantly predict PSMU among (young) adults, whereas Paakkari et al. (2021) found that PSMU was more prevalent in older adolescents compared with younger adolescents [12,19]. The same study found that there was no association between family affluence and PSMU [19]. Findings on the relationship between gender and PSMU are varied; Bányai et al. (2017) and Kircaburun et al. (2019) found that girls are at a higher risk of PSMU than boys, whereas Paakkari et al. (2021) did not find gender differences for PSMU [2,17,19]. With reference to the Bio-Ecological Model, we also want to explore if migration background influences PSMU among adolescents. Previous research has indicated that online media have become an important method of transnational communication, especially among younger migrants [27]. This is particularly relevant as the data for this study originate from Luxembourg, where approximately 47% of the population had a non-Luxembourgish nationality in 2020 [28]. Due to contradicting results and a general lack of research, we explore the following research question:

**Research Question 1:** *Which sociodemographic factors contribute to the risk of problematic social media use among adolescents?*

**Social Support Factors**

That close relationships have an influence on adolescents responsiveness to media, activities and overall development is in line with both the DSMM and the Bio-Ecological Model. In a large cross-national study, Boer et al. (2020) found that problematic social media users reported lower levels of family and peer support in all participating countries [18]. Kircaburun et al. (2019) found that low levels of general belongingness is a direct predictor of PSMU and cyberbullying perpetration, and that low social connectedness indirectly predicted PSMU and cyberbullying perpetration through mediation with depression [2]. Several cross-sectional and longitudinal studies found associations between cyberbullying victimization and PSMU or problematic internet use [29,30], as well as for cyberbullying perpetration, indicating a relation between the two online problem behaviors [2]. We explore these relationships for PSMU as well. As most findings indicate that lower social support is a risk factor for problematic social media use, we hypothesize that:

**Hypothesis** **1:** *Adolescents who indicate lower levels of social support from their parents, peers and teachers have an increased risk of problematic social media use*.

**Hypothesis** **2:** *Adolescents who indicate that they are perpetrators or victims of cyberbullying have an increased risk of problematic social media use*.

**Well-Being Factors**

Well-being is at the center of the Bio-Ecological Model. Previous studies found associations between problematic social media use and well-being, indicating that adolescents with PSMU indicated lower levels of life satisfaction, increased depressive mood and more health complaints compared with adolescents who did not have PSMU [12,18,19,31]. A possible explanation for this behavior is that negative emotional states can influence addictive behaviors, such as PSMU [22]. Accordingly, individuals may attempt to increase their well-being through increased social media use. We hypothesize that lower levels of life satisfaction, higher levels of perceived stress and more psychosomatic complaints increase the susceptibility for problematic social media use among adolescents:

**Hypothesis** **3:** *Adolescents with lower levels of well-being have an increased susceptibility for problematic social media use*.

**Media Use Factors**

A range of studies has explored how different ways of using social media can influence PSMU or its overarching construct, problematic internet use. It is important to differentiate between intense social media use and PSMU [21]. A large cross-national study by Boer et al. (2020) showed that the association between the intensity of social media use and well-being for adolescents varied between countries and indicators of mental well-being, whereas PSMU was consistently associated with lower well-being in all countries [18]. Contrarily, a study by Savci et al. (2020) using machine learning found that the frequency of daily social media use and checking social media accounts is an important predictor of PSMU among university students [32]. Motives for social media use, such as using social media to pass time, have been associated with PSMU as well [33]. A preference for online social interaction (POSI), which refers to a preference for online communication over face-to-face communication based on increased feelings of safety, confidence and efficacy, has been identified as an important component of problematic internet use that can predict negative outcomes of problematic use [34,35]. In addition, Moretta and Buodo (2018) found that POSI predicted the use of Facebook for mood regulation purposes and the impaired self-regulation of Facebook use, and indirectly contributed to negative outcomes of problematic Facebook use [36]. Accordingly, we hypothesize that POSI increases the risk of PSMU. In addition, based on the mixed findings and general lack of research regarding the relationship between the intensity of electronic media communication and PSMU among adolescents, we phrase the following research question:

**Research Question 2:** *Is a higher level of intensity of electronic media a risk factor for problematic social media use among adolescents?*

**Hypothesis** **4:** *Adolescents with higher levels of preference for online social interaction have an increased susceptibility for problematic social media use*.

## 2. Methods

### 2.1. Participants and Procedure

The Health Behavior in School-aged Children (HBSC) study is a WHO collaborative cross-national survey study that was conducted every four years across 50 countries and regions in Europe, Asia and North America. The data for this study were derived from the HBSC Luxembourg study in 2018. The HBSC Luxembourg study collected nationally representative data from students aged 11–18 years old. Primary and secondary school classes were selected at random as primary sampling units. Schools that did not teach according to the national curriculum and special needs schools were excluded from sampling. Invited students and their guardians received a letter to inform them about the study, as well as a consent form. Students who had given consent were included in the study. Teachers reminded the students of their right to decline participation in the study at any time. Data were collected using paper–pencil questionnaires. The original HBSC questionnaires were developed in English. The questions were then translated to the respective language for the participating countries, and back translated to ensure that the original and translated questions matched. The HBSC Luxembourg study included questions in both German and French, as these were the main written languages in schools. A total of 8687 students aged 11–18 participated in the 2018 survey of the HBSC study in Luxembourg. A flowchart of the study participants can be found in Figure S1 in the supplementary material. Ethical approval was granted by the *Comité National d’Ethique de Recherche* (CNER, Avis N°201711/02) and the Ethics Review Panel of the University of Luxembourg (ERP 17-059 HBSC 2018). In addition, the *Commission nationale pour la protection des données* was informed about the study.

### 2.2. Measures

#### 2.2.1. Media Use Factors

The outcome variable problematic social media use was assessed using the Social Media Disorder Scale, which was developed by van den Eijnden et al. [21]. The scale is based on shared characteristics between social media platforms and is, thus, not limited to one platform, which is important for the progress of research on social media addiction as the platforms are continuously evolving and being replaced. In this study, the short 9-item scale was used. The items reflected diagnostic criteria for problematic social media use, which were based on the criteria for Internet Gaming Disorder as described in the DSM-5. For example, adolescents were asked whether in the past year they regularly could not think about anything else but using social media again, tried to spend less time on social media but failed or neglected other activities in order to use social media. The two response options were yes and no. A continuous scale ranging from 0 to 9 was computed by counting up the positive answers. The internal consistency of the scale in this sample was satisfactory (Cronbach’s alpha = 69). Intensity of electronic media communication was measured using four items asking adolescents how often they had online contact with close friends, friends from a larger friend group, friends met through the internet and other people such as parents, siblings or teachers. Two of the items were adapted from the EU Kids Online study and Net Children Go Mobile study, and two additional items were added by the HBSC study [37]. The six response options ranged from *never to almost never* to *almost all the time throughout the day*, and included a *don’t know / does not apply* category, which was excluded when computing the final score for the scale. A continuous scale variable ranging from 1 (low intensity) to 5 (high intensity) was created by computing a mean score of the four items. Preference for online social interaction (POSI) was assessed using a 3-item form adapted from the 5-item scale perceived depth of communication originally developed by Valkenburg and Peter [38]. The adolescents were asked to what extent they agreed with the statements that on the internet they could talk easier about secrets, feelings and concerns than in a face-to-face encounter. The five response options ranged from *strongly disagree* to *strongly agree*. A continuous scale ranging from 1 (low POSI) to 5 (high POSI) was created by averaging the answer scores of the three items.

#### 2.2.2. Well-Being Factors

Psychological stress was measured using the short form Cohen Perceived Stress Scale [39]. The adolescents were asked how often in the last month they felt unable to control the important things in their life, confident about their ability to handle their personal problems, felt that things were going their way and felt that difficulties were piling up so high that you could not overcome them. The five response options ranged from *never* to *very often*. The responses were used to create a continuous scale ranging from 0 (low level of stress) to 16 (high level of stress). Subjective well-being (life satisfaction) was assessed by asking the adolescents to place themselves anywhere on a Cantril ladder where a score of 0 represented the worst possible life, and a score of 10 the best possible life [40]. Psychosomatic complaints were assessed using the HBSC symptom checklist, developed by Haugland and Wold for the HBSC study [41]. The adolescents were asked how often in the last six months they had a headache, stomachache, backache, felt low, were irritable, felt nervous or had difficulties falling asleep. The five response options ranged from *rarely or never (0)* to *about every day (4).* A continuous scale was created by summing up the responses, resulting in an index ranging from 0 (no health complaints) to 32 (high level of health complaints).

#### 2.2.3. Social Support Factors

Family support was assessed using the family subscale of the Multidimensional Scale of Perceived Social Support (MSPSS) [42,43]. The adolescents were asked to what extent they agreed that their family really tries to help them, that they obtain emotional help and support from their family, that they can talk about their problems with their family, and that their family is willing to help them make decisions. The seven response options ranged from *very strongly disagree* to *very strongly agree*. A scale ranging from 1 (low level of family support) to 7 (high level of family support) was obtained by calculating the average from the responses to the four items. Peer support was assessed using the friend subscale of the MSPSS [42,43]. The adolescents were asked to what extent they agreed that their friends really try to help them, that they can count on their friends when things go wrong, that they can share joys and sorrows with their friends and that they can talk about their problems with their friends. The seven response options ranged from *very strongly disagree* to *very strongly agree*. A scale ranging from 1 (low level of peer support) to 7 (high level of peer support) was obtained by calculating the average from the responses to the four items. Support from teachers was assessed by asking the adolescents to what extent they agreed that their teachers accept them as they are, care about them as a person and whether they feel trust in their teachers. There were five response options ranging from *strongly agree* to *strongly disagree*. A scale ranging from 1 (low level of teacher support) to 5 (high level of teacher support) was obtained by calculating the average from the responses to the three items. The items of this scale were developed by the HBSC study and have been used since the 2009/10 survey wave [44]. Cyberbully victimization was assessed by asking adolescents how often they were cyberbullied in the last months. The five response options ranged from *I have not been cyberbullied* to *several times a week*. The possible responses formed a scale ranging from one (not a victim of cyberbullying) to five (victim of cyberbullying frequently). The items on cyberbully victimization were adapted to cyberbullying from items in the (Revised) Bully / Victim Questionnaire originally developed by Olweus [45,46]. Cyberbully perpetration was assessed by asking adolescents how often they cyberbullied another person in the last months. The five response options ranged from *I have not cyberbullied another person* to *several times a week*. The possible responses form a scale from one (not a cyberbully perpetrator) to five (cyberbullies other frequently). The items on cyberbullying perpetration were adapted to cyberbullying from items in the (Revised) Bully / Victim Questionnaire originally developed by Olweus [45,46].

#### 2.2.4. Sociodemographic Factors

Gender was determined by asking the adolescents whether they are a girl or a boy. For the purpose of this study, an indicator variable was created using the boys as the reference category. The age of the adolescents was obtained by asking the adolescents for their year and month of birth. Relative family affluence was assessed using the Family Affluence Scale III (FAS). An index of FAS was created with responses to six items that reflected wealth (ownership of cars, computers, dishwasher, bathrooms, having an own bedroom and travelling abroad). This scale was developed by the HBSC study as a proxy for socioeconomic status. A relative measure of family affluence was obtained by transforming the absolute affluence scores to ridit scores, resulting in a scale ranging from zero to one [47]. Migration background was obtained by asking the adolescents in which country they themselves, their mother and their father were born. Using the responses to these questions, adolescents were categorized into three groups: *native*, *first-generation immigrant* or second-*generation immigrant*. This categorization has been used frequently in HBSC publications [48]. Indicator variables were created using the native category as the reference category.

### 2.3. Data Analysis

A hierarchical multiple regression analysis was conducted to identify predictors of PSMU [49]. The predictors were added to the regression model in four blocks. Model 1 included the sociodemographic factors age, gender, family affluence and migration background. In Model 2, social support factors family support, peer support, teacher support, cyberbully victimization and cyberbully perpetration were added. Model 3 included well-being factors life satisfaction, psychosomatic complaints and stress. Finally, in Model 4, media use factors the intensity of online communication and preference for online social interaction were added to the model. Missing data were handled using listwise deletion. In addition, index scores for the various scales were not computed if the adolescents indicated that the items were not applicable to them, or if they had too many missing values on scales with multiple answers. This brought the number of participants included in the analysis to *N* = 5794. The significance level for the analysis was set at *p* ≤ 0.05. The data were weighted by the distribution of adolescents across school grades. Accordingly, the distribution of the adolescents across school grades in the weighted sample was in line with the distribution in the population as a whole. All analyses were carried out using IBM SPSS version 25.

## 3. Results

### 3.1. Descriptive Statistics

When the dichotomous version of the problematic social media use scale was used, applying the recommended cut-off of six symptoms for problematic use, the prevalence of problematic social media use in the sample was 5.9%. There was a higher prevalence of PSMU among girls (6.7%) compared with boys (4.9%). A description of the sample can be found in Table 1. The bivariate associations between the factors included in the model were obtained using a correlation matrix, which can be found in Table S1 in the Supplementary Materials.

### 3.2. Hierarchical Regression

A hierarchical linear regression analysis was performed to identify predictors of problematic social media use among adolescents in Luxembourg. The results of the analysis can be found in Table 2. The analyses were also conducted separately for girls and boys, the results of which can be found in Table S2 and S3 in the Supplementary Materials. In Model 1, all included sociodemographic predictors significantly contributed to the explanation of the variance in PSMU. In Model 2, all included sociodemographic and social support predictors, with the exception of family affluence and peer support, significantly contributed to the explanation of PSMU. In Model 3, of the previously included sociodemographic and social support factors, family affluence, peer support and teacher support were no longer significant contributors to the model. Of the newly included well-being factors, predictors perceived stress and psychosomatic health complaints significantly contributed to the explanation of PSMU, whereas life satisfaction did not. In Model 4, the majority of predictors significantly contributed to the explanation of the variance in PSMU, with the exception of family affluence, teacher support and life satisfaction.

The standardized beta coefficients could be used to interpret the relative importance of the predictors in the final model [49]. Where sociodemographic factors were concerned, age was the most important predictor. There was a negative relationship between age and problematic social media use, meaning that PSMU generally decreases with age. Cyberbully perpetration was the most important predictor within the social support factors block. As cyberbullying perpetration increased, so did the risk of PSMU. Both perceived stress and psychosomatic health complaints were important predictors within the well-being factors block and for the model as a whole. Both predictors had a positive relationship with PSMU, indicating that as stress and psychosomatic health complaints increased, so did the risk of PSMU. Both media use factors, POSI and the intensity of electronic media communication, were important predictors in the final model, and had a positive relationship with PSMU.

All four models significantly contributed to the explanation of the variance in PSMU. Accordingly, the null hypothesis that the respective model did not contribute to the explanation of PSMU was rejected. The first model with the sociodemographic factors accounted for 3.3% of the variance in PSMU. The second model, in which the social support factors were added, explained 9.8% of the variance. The third model included the well-being factors, which explained 15.3% of the variance in PSMU. The fourth and final model, in which the media use factors were added, explained 22.3% of the variance in PSMU. The adjusted R^2^ changed indicated that the inclusion of the social support factors and media use factors contributed the largest increase in explained variance in PSMU.

## 4. Discussion

An increasing number of adolescents develop a problematic pattern of social media use. According to the present findings, the prevalence of PSMU among adolescents aged 11–18 years old in Luxembourg was 5.9%. Adolescents’ (problematic) social media use can be examined using the DSMM [5] and the Bio-Ecological Model [15], both of which consider the role of social relationships and individual-level variables in shaping the behavior and development of adolescents.

### 4.1. Sociodemographic Factors and PSMU

Gender, age and migration status significantly contributed to the final model, which answered research question one. However, sociodemographic factors were not key predictors for the explanation of variance in PSMU when compared with the variance explained by the addition of the other blocks. This was coherent with the results of previous studies [12,33,50]. PSMU was negatively related with age in this study, which was consistent with findings of a study that was conducted in a highly similar age group that also used the Social Media Disorder Scale [51]. However, these results were in contradiction with other studies [12,19]. The results also indicated that a migration background was predictive of PSMU. As social media are a popular way to communicate with friends and family in the country of origin [27], migrants might be at an increased risk of developing PSMU. This result was particularly relevant for Luxembourg, where a vast percentage of the population has a migration background, and should be considered in efforts to prevent PSMU in Luxembourg. Although not an important direct predictor of PSMU, gender nonetheless plays an important role in shaping social media behavior as boys and girls tend to have different motives and purposes for using social media, and display different types of internet use in general [12,52,53]. Gender can, as such, influence the behavior adolescents engage in on social media and, through this, their susceptibility to media effects can be affected.

### 4.2. Social Support and PSMU

The results indicated partial support for hypothesis one, as there was a negative relationship between PSMU and parental and peer support. This was coherent with the results from a large cross-national study [18], and fit with the proposition from the DSMM and Bio-Ecological Model about the importance of social relationships in shaping adolescents (online) behavior. The increased risk of PSMU could potentially be explained by a motivation to seek support online, in a space where adolescents feel more comfortable socializing with other people [54].The study also found that cyberbullying perpetration and victimization were predictors of PSMU, confirming hypothesis two. This was in accordance with results from previous studies on the relationship between cyberbullying and problematic internet use, the proposed overarching construct of problematic social media use [29,30]. A longitudinal study found that cyberbullying victimization predicted an increase in problematic internet use among adolescents, whereas this relationship was not found in the inverse direction [30]. Using the internet or social media to escape from negative feelings is an important component of PSMU, the Social Media Disorder Scale and problematic internet use [21,55]. The explanation offered for this increased risk of problematic internet use, which may also help explain the increased risk of PSMU in this study, is that victims of cyberbullying use the internet as a means to escape from the distress caused by cyberbullying [30]. Furthermore, cyberbullying perpetration was associated with PSMU in previous research [2]. The authors indicated that adolescents who feel socially disconnected from their surroundings may compensate a need for connection and socializing by spending a disproportionate amount of time on social media and engage in antisocial behaviors if they feel rejected in online settings as well. The results of this study and previous studies are in line with the proposition that problematic behaviors in the online context are related [2,56].

### 4.3. Well-Being and PSMU

Perceived stress and psychosomatic health complaints contribute significantly to the explanation of the variance in PSMU in the final model, which provided partial support for hypothesis three. Perceived stress was also identified as a predictor of PSMU for children and adolescents in a recent study that used the same instruments to measure PSMU and stress as the present study [51]. Adolescents who experience high levels of stress may regulate stress-related emotions by turning to social media, applying a strategy called emotion-focused coping [51,57]. In addition, experiencing psychosomatic complaints was a significant predictor of PSMU in this study. This was coherent with a previous study that found a higher prevalence of multiple psychosomatic complaints among adolescents with PSMU compared with adolescents that were not or moderately at risk for PSMU [19]. Life satisfaction was not a significant predictor of PSMU in this study. Previous studies report mixed results for this relationship, and making comparisons between studies is difficult due to varying age groups and instruments [22,58,59]. The results could imply that PSMU is differently related to the various aspects of well-being. In support of this, a recent study found that positive and negative effects were important predictors of PSMU in an adult sample, whereas psychological well-being was not [12]. It would be important to explore how different aspects of well-being impact adolescents’ susceptibility for PSMU in further studies.

### 4.4. Media Use and PSMU

This study found that POSI is an important predictor of PSMU, which confirmed hypothesis four. These findings were consistent with the cognitive behavioral model of the Generalized Problematic Internet use proposed by Caplan (2010), which builds on the original model proposed by Davis (2001), and prior research on POSI [34,35,60,61]. Individuals experiencing psychosocial distress may develop POSI because they feel that online interaction is less threatening than face-to-face interaction, and gives them more control over self-presentation. POSI is an important predictor for problematic internet use, both directly and indirectly through mood-regulation [35]. Previous research confirmed this model for problematic internet and Facebook use in adolescent and young adult samples [36,62]. The present study provided initial support for the hypothesis that POSI is an important predictor of PSMU in an adolescent sample, although further research on the relationships between POSI, using social media for mood regulation and PSMU is necessary. These results suggested that prohibiting the development of POSI could be important in efforts to prevent PSMU. The results also indicated that a higher level of intensity of electronic media communication is predictive of PSMU among adolescents, which answered research question two. This was in line with previous studies which found that the overall time spend on social media and the frequency of checking social media accounts are important predictors of PSMU in adolescents and young adults [31,32]. These findings make sense intuitively, as the Social Media Disorder scale is correlated with social media use behavior such as frequency of daily use [21,32]. However, it is important to note that time-intensive or highly engaged social media use is not immediately a cause for concern [31], supported by the finding that POSI and the intensity of electronic media communication explained approximately 7% in the variance of PSMU. POSI and the intensity of electronic media communication are of higher relative importance for PSMU than other predictors in the model, as indicated by the standardized beta coefficients. Previous research has also highlighted the importance of other factors of social media use behavior for the development and severity of PSMU, such as the fear-of-missing-out and motives for social media use, in populations varying in age [12,32,33].

#### 4.4.1. Limitations and Contributions

This study had a number of potential limitations that should be considered. First, the selected predictors in this study were able to explain approximately 22% of the variance in PSMU among adolescents in Luxembourg. Whereas this left a moderate amount of variance left to explain, similar studies that aimed to identify predictors of PSMU using a hierarchical regression analysis obtained a comparable percentage of explained variance by the predictors included in the models [12,33,51]. The inclusion of more predictors related to social media use behavior could potentially improve the explained variance in PSMU. Motives for social media use, which are rooted in the uses and gratifications theory by Katz et al. (1973), were identified as an important predictor of PSMU in multiple studies [12,13,33]. Motives such as alleviating boredom and socializing with new and existing relations in particular were found to contribute to PSMU [12,13,33]. Accordingly, the current study could have benefitted from the inclusion of motives for social media use in the model. Second, this study did not consider the role of the social media content, which has the potential to influence the responses to social media, which then influence media effects [5]. The impact of social media content on the development of PSMU, and the active role that adolescents have in this process by selecting content, has remained largely unexplored and should be considered in future studies [5]. Third, this study used cross-sectional data, meaning that the causality between the predictors and PSMU could not be inferred. Accordingly, it is possible that the effect of interest goes in the other direction, meaning that PSMU could influence well-being, for example. Further research should employ a longitudinal design. Nonetheless, modern media effect theories recognize that media effects are bidirectional. Accordingly, media use can cause changes in media users, and media effects themselves can influence media use, media responses and differential susceptibility variables as proposed by the DSMM [5]. Fourth, the results of this study cannot be generalized to adolescents in countries other than Luxembourg.

Despite the limitations noted above, this study determined a few meaningful contributions to the current knowledge on predictors of PSMU in adolescents. To the best of the authors knowledge, this was the first study to examine predictors of PSMU in adolescents in Luxembourg. Investigating predictors of PSMU in different countries is important, as previous studies found cross-national differences in the prevalence of PSMU and problematic internet use [18,63]. In addition, this study used a nationally representative sample for adolescents, which meant that the findings of the study could be generalized to the adolescent population in Luxembourg. Furthermore, the importance of this study was that it was one of the first to incorporate a wide range of predictors of PSMU in adolescents, combining sociodemographic factors, social support factors, well-being and media use factors in one comprehensive model. This allowed for the comparison of the importance of the predictors where PSMU was concerned. In addition, as the predictors were selected based on theoretical frameworks, the results provide support for some of the propositions of the DSMM and the Bio-Ecological Model, and its relevance for adolescent social media use. Lastly, the knowledge obtained from this study furthered our current understanding of factors associated with PSMU in adolescents. This knowledge can be used to inform further research on predictors for PSMU in other countries, and could contribute to the prevention of PSMU in adolescents both nationally and internationally.

#### 4.4.2. Theoretical Implications

This study identified several predictors of PSMU related to sociodemographic factors, social support, well-being and media use in an adolescent population, which indicated that there is a wide range of predictors that influence adolescents’ susceptibility to PSMU. That these predictors were diverse was in accordance with the DSMM and the Bio-Ecological Model. The results indicated that the social relationships of adolescents and the interaction with their environment are important factors to consider in their susceptibility for PSMU, which was coherent with the influence of the microsystem on adolescents’ behavior proposed by the Bio-Ecological Model. Therefore, increasing feelings of perceived social support and reducing cyberbullying perpetration among adolescents may help to reduce PSMU by decreasing the need of adolescents to seek social support online and escape from negative feelings by using social media. The results also suggested that different aspects of well-being and media use are significant predictors of PSMU in adolescents. This was in line with the DSMM, as differential susceptibility variables such as perceived stress and POSI could influence adolescents’ susceptibility for PSMU. Subsequently, inhibiting POSI and increasing the well-being of adolescents may also be effective ways of preventing PSMU. Taken together, the results of the study underlined the complexity that underlies PSMU and the diverse range of factors that are linked to its development.

## 5. Conclusions

This study determined a number of relevant contributions to the limited knowledge on predictors of PSMU in adolescents. The results indicated that, in particular, cyberbullying perpetration, stress, psychosomatic health complaints, POSI and the intensity of electronic media communication are important factors for the development of PSMU in adolescents. In addition, the results suggested that sociodemographic factors are not key predictors of PSMU. The findings were in accordance with the propositions determined by the Differential Susceptibility to Media Effects Model and the Bio-Ecological Model. Accordingly, interventions that aim to prevent or reduce PSMU in adolescents need to go beyond reducing the frequency and intensity of social media use and should consider the diverse range of predictors related to the development of PSMU, as well as the influence of the relationships and environment on the behavior of adolescents.

## Figures and Tables

**Table 1:** Prevalence of sociodemographics, social support, well-being, media use and problematic social media use (PSMU) in the non-weighted sample.

Variable	Statistics
Gender	Girl = 4348 (50.1%) Boy = 4328 (49.9%)
Age	11–18 age range, $\bar{x}$ = 14.38, σ = 2.16
Family affluence	0–1 range, $\bar{x}$ = 0.50, σ = 0.28
Migration background	Native = 2487 (28.8%) 1st generation immigrant = 1915 (22.2%) 2nd generation immigrant = 4232 (49.0%)
Parent support	1–7 range, $\bar{x}$ = 5.71, σ = 1.61
Peer support	1–7 range, $\bar{x}$ = 5.65, σ = 1.57
Teacher support	1–5 range, $\bar{x}$ = 2.46, σ = 0.92
Cyberbully victimization	1–5 range, $\bar{x}$ = 1.13, σ = 0.50
Cyberbully perpetration	1–5 range, $\bar{x}$ = 1.16, σ = 0.56
Psychological stress	0–16 range, $\bar{x}$ = 6.87, σ = 2.97
Life satisfaction	0–10 range, $\bar{x}$ = 7.56, σ = 1.81
Psychosomatic complaints	0–32 range, $\bar{x}$ = 10.01, σ = 6.3
Preference for online social interaction	1–5 range, $\bar{x}$ = 2.06, σ = 1.10
Intensity of electronic media communication	1–5 range, $\bar{x}$ = 2.96, σ = 0.92
PSMU	0–9 range, $\bar{x}$ = 1.92, σ = 1.95 Problematic use = 415 (5.9%) Non-problematic use = 6642 (94.1%)

Note: $\bar{x}$ = mean, σ = standard deviation

**Table 2:** Results of the hierarchical linear regression, predictors of PSMU.

	Model 1		Model 2		Model 3		Model 4	
	B	β	B	β	B	β	B	β
**Step 1: Sociodemographic factors**								
Gender (reference = boy)	0.407	0.106 ***	0.375	0.098 ***	0.126	0.033*	0.184	0.048 ***
Age	−0.069	−0.076 ***	−0.094	−0.104 ***	−0.101	−0.112 ***	−0.111	−0.122 ***
Family affluence	−0.251	−0.037 **	−0.153	−0.022	−0.061	−0.009	−0.093	−0.014
1st generation migrant	0.564	0.119 ***	0.508	0.107 ***	0.490	0.103 ***	0.438	0.093 ***
2nd generation migrant	0.404	0.105 ***	0.348	0.091 ***	0.329	0.086 ***	0.283	0.074 ***
**Step 2: Social support factors**								
Parent support			−0.149	−0.121 ***	−0.059	−0.048 **	−0.043	−0.035 *
Peer support			−0.015	−0.011	0.017	0.013	−0.034	−0.026 *
Teacher support			0.154	0.071 ***	0.049	0.023	0.052	0.024
Cyberbully victimization			0.343	0.090 ***	0.219	0.057 ***	0.129	0.034 **
Cyberbully perpetration			0.577	0.134 ***	0.553	0.129 ***	0.482	0.112 ***
**Step 3: Well-being factors**								
Stress					0.095	0.147 ***	0.082	0.127 ***
Life satisfaction					0.001	0.001	0.001	0.001
Psychosomatic complaints					0.052	0.168 ***	0.043	0.138 ***
**Step 4: Media use factors**								
Preference for online social interaction							0.357	0.206 ***
Intensity of electronic media communication							0.330	0.153 ***
F	40.16 ***		84.5 ***		126.9 ***		261.3 ***	
R	0.183		0.315		0.394		0.474	
Adjusted R^2^	0.033		0.098		0.153		0.223	
∆R^2^	0.033		0.066		0.056		0.070	

Note: * *p* ≤ 0.05; ** *p* ≤ 0.01; *** *p*≤ 0.001.

## Data Availability

The data presented in this study are available on request from the corresponding author. The data are not publicly available due to an embargo.

## Supplementary Materials

A correlation matrix of the variables included in the regression model is available online https://www.mdpi.com/article/10.3390/ijerph182211878/s1. Table S1: correlation matrix of the variables included in the hierarchical regression analysis. Table S2: Results of the hierarchical linear regression, predictors of PSMU, for girls (N = 3044). Table S3: Results of the hierarchical linear regression, predictors of PSMU, for boys (N = 2750). Figure S1: Flowchart of the study participants
