# Supplementary material for: Predictors of Problematic Social Media Use in a Nationally Representative Sample of Adolescents in Luxembourg
Source: Int J Environ Res Public Health. 2021 Nov 12;18(22):11878. doi: 10.3390/ijerph182211878 (PMC8619406; doi:10.3390/ijerph182211878)
Supplement: Supplementary file 1 [file ijerph-18-11878-s001.zip › Table S1 - Correlation matrix.pdf]

Table S1: Correlation matrix of the variables included in the hierarchical regression analysis

Preference for online social interaction	Psychosomatic complaints	Life satisfaction	Stress	Cyberbully perpetration	Cyberbully victimisation	Teacher support	Peer support	Parent support	2nd generation migrant	1st generation migrant	Age	Family affluence	Gender	PSMU	
0.292 ***	0.282 ***	-0.176 ***	0.281 ***	0.178 ***	0.167 ***	0.104 ***	-0.072 ***	-0.163 ***	0.053 ***	0.070 ***	-0.079 ***	-0.063 ***	0.106 ***		PSMU
-0.009	0.282***	-0.070***	0.169***	-0.068***	0.050***	0.069***	0.132***	-0.079***	0.023*	-0.026*	-0.001	-0.011		0.106***	Gender
-0.031**	-0.042**	0.200***	-0.133***	-0.003	-0.053***	0.030*	0.118***	0.110***	-0.035**	-0.195***	-0.019		-0.011	-0.063***	Family affluence
0.034**	0.114***	-0.129***	0.043**	0.006	-0.041**	0.215***	0.052***	-0.131***	-0.047***	0.013		-0.019	-0.001	-0.079***	Age
0.001	-0.002	-0.068***	0.027*	0.048***	0.025*	-0.022*	-0.045***	0.000	-0.498***		0.013	-0.195***	-0.026*	0.070***	1st generation migrant
0.029*	0.028*	-0.052***	0.043***	-0.011	0.006	0.026*	-0.034**	-0.056***		-0.498***	-0.047***	-0.035**	0.023*	0.053***	2nd generation migrant
-0.154***	-0.304***	0.419***	-0.366***	-0.066***	-0.111***	-0.259***	0.276***		-0.056***	0.000	-0.131***	0.110***	-0.079***	-0.163*	Parent support
-0.035**	-0.102***	0.200***	-0.197***	-0.058***	-0.108***	-0.089***		0.276***	-0.034**	-0.045***	0.052***	0.118***	0.132***	-0.072***	Peer support
0.059***	0.249***	-0.236***	0.244***	0.099***	0.037**		-0.089***	-0.259***	0.026*	-0.022*	0.215***	0.030*	0.069***	0.104***	Teacher support
0.126***	0.166***	-0.145***	0.158***	0.345***		0.037**	-0.108***	-0.111***	0.006	0.025*	-0.041**	-0.053***	0.050***	0.167***	Cyberbully victimisation
0.094***	0.079***	-0.074***	0.071***		0.345***	0.099***	-0.058***	-0.066***	-0.011	0.048***	0.006	-0.003	-0.068***	0.178***	Cyberbully perpetrator
0.168***	0.521***	-0.467***		0.071***	0.158***	0.244***	-0.197***	-0.366***	0.043***	0.027*	0.043**	-0.133***	0.169***	0.281***	Perceived stress
-0.129***	-0.397***		-0.467***	-0.074***	-0.145***	-0.236***	0.200***	0.419***	-0.052***	-0.068***	-0.129***	0.200***	-0.070***	-0.176***	Life satisfaction
0.165***		-0.397***	0.521***	0.079***	0.166***	0.249***	-0.102***	-0.304***	0.028*	-0.002	0.114***	-0.042**	0.282***	0.282***	Psychosomatic complaints
	0.165***	-0.129***	0.168***	0.094***	0.126***	0.059***	-0.035**	-0.154***	0.029*	0.001	0.034**	-0.031**	-0.009	0.292***	Preference for online social interaction
0.139***	0.052***	0.019	0.007	0.060***	0.048***	0.019	0.198***	0.058***	0.004	0.030*	0.065***	0.042***	0.031**	0.188***	Intensity of electronic media communication

Intensity of electronic media communication	0.188 ***	0.031**	0.042**	0.065***	0.003*	0.004	0.058***	0.198***	0.019	0.048***	0.060***	0.007	0.019	0.052***	0.139***	

* $p \leq 0.05$ ** $p \leq 0.01$ *** $p \leq 0.001$
