# Supplementary material for: Predictors of Problematic Social Media Use in a Nationally Representative Sample of Adolescents in Luxembourg
Source: Int J Environ Res Public Health. 2021 Nov 12;18(22):11878. doi: 10.3390/ijerph182211878 (PMC8619406; doi:10.3390/ijerph182211878)
Supplement: Supplementary file 1 [file ijerph-18-11878-s001.zip › Table S2 and Table S3.pdf]

Table S2: Results of the hierarchical linear regression, predictors of PSMU, for girls (N=3044)

	Model 1		Model 2		Model 3		Model 4	
	B	β	B	β	B	β	B	β
**Step 1: Sociodemographic factors**								
Age	-0.053	-0.057***	-0.089	-0.097***	-0.112	-0.121***	-0.117	-0.127***
Family affluence	-0.146	-0.021	-0.020	-0.003	0.086	0.013	0.080	0.012
1st generation migrant	0.527	0.108***	0.499	0.102***	0.461	0.095***	0.461	0.095***
2nd generation migrant	0.392	0.101***	0.312	0.081***	0.311	0.080***	0.298	0.077***
**Step 2: Social support factors**								
Parent support			-0.182	-0.155***	-0.071	-0.060**	-0.048	-0.041*
Peer support			-0.004	-0.003	0.038	0.028	-0.008	-0.06
Teacher support			0.235	0.106***	0.116	0.052**	0.103	0.047**
Cyberbully victimisation			0.353	0.094***	0.196	0.052**	0.111	0.030
Cyberbully perpetration			0.813	0.154***	0.790	0.149***	0.693	0.131***
**Step 3: Well-being factors**								
Stress					0.101	0.156	0.081	0.125***
Life satisfaction					-0.025	-0.023	-0.031	-0.029
Psychosomatic complaints					0.050	0.164***	0.042	0.136***
**Step 4: Media use factors**								
Preference for online social interaction							0.358	0.202***
Intensity of electronic media communication							0.335	0.149***
F	12.75***		62.73***		75.94***		129.77***	
R	0.128		0.330		0.413		0.486	
Adjusted R^2	0.017		0.109		0.171		0.236	
ΔR^2	0.017		0.092		0.062		0.065	

* p ≤ 0.05 ** p ≤ 0.01 *** p≤ 0.001

Table S3: Results of the hierarchical linear regression, predictors of PSMU, for boys (N=2750)

	Model 1		Model 2		Model 3		Model 4	
	B	β	B	β	B	β	B	β
**Step 1: Sociodemographic factors**								
Age	-0.086	-0.099***	-0.099	-0.113***	-0.089	-0.102***	-0.102	-0.117***
Family affluence	-0.370	-0.055**	-0.305	-0.046*	-0.229	-0.034	-0.286	-0.043*
1st generation migrant	0.602	0.133***	0.532	0.117***	0.530	0.117***	0.422	0.093***
2nd generation migrant	0.422	0.112***	0.390	0.104***	0.356	0.095***	0.274	0.073***
**Step 2: Social support factors**								
Parent support			-0.095	-0.074***	-0.033	-0.025	-0.025	-0.020
Peer support			-0.035	-0.027	-0.014	-0.011	-0.068	-0.053**
Teacher support			0.066	0.032	-0.017	-0.008	0.003	0.001
Cyberbully victimisation			0.321	0.083***	0.236	0.061**	0.148	0.038*
Cyberbully perpetration			0.473	0.131***	0.434	0.120***	0.371	0.103***
**Step 3: Well-being factors**								
Stress					0.086	0.130***	0.080	0.121***
Life satisfaction					0.028	0.026	0.033	0.030
Psychosomatic complaints					0.053	0.155***	0.044	0.128***
**Step 4: Media use factors**								
Preference for online social interaction							0.354	0.211***
Intensity of electronic media communication							0.321	0.159***
F	22.11***		28.37***		49.25***		128.71***	
R	0.177		0.281		0.355		0.449	
Adjusted R^2	0.030		0.076		0.122		0.197	
ΔR^2	0.031		0.048		0.047		0.075	

* $p \leq 0.05$ ** $p \leq 0.01$ *** $p \leq 0.001$
