# Supplementary figures and images for: Predictors of Problematic Social Media Use in a Nationally Representative Sample of Adolescents in Luxembourg
Source: Int J Environ Res Public Health. 2021 Nov 12;18(22):11878. doi: 10.3390/ijerph182211878 (PMC8619406; doi:10.3390/ijerph182211878)

**Figure S1:** Flowchart of the study participants

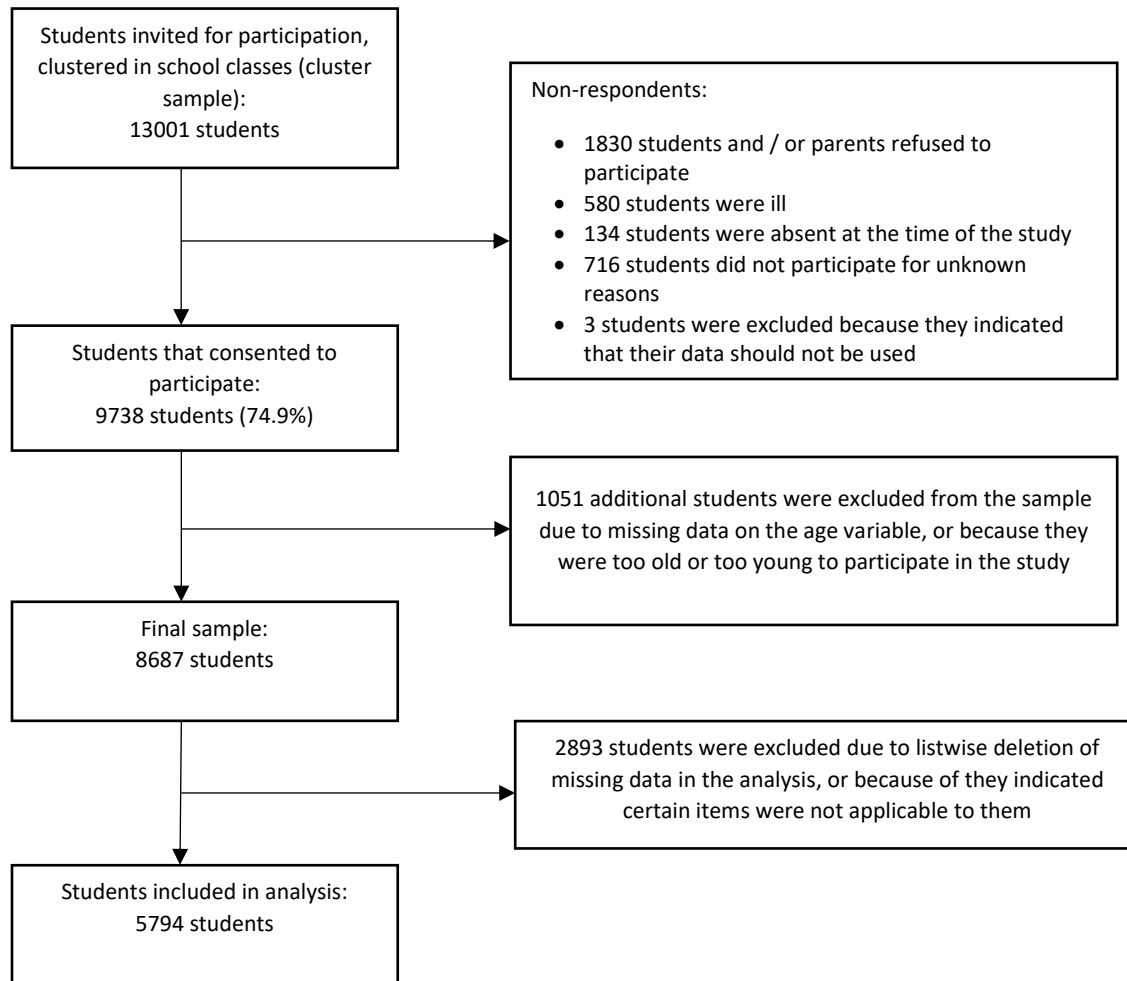

Supplement: Supplementary file 1 [file ijerph-18-11878-s001.zip › Figure S1 - Flowchart of the study participants.pdf]
